# Physical and mental determinants of dropout and retention among nursing students: protocol of the SPRiNG cohort study

**DOI:** 10.1186/s12912-018-0296-9

**Published:** 2018-06-22

**Authors:** Ellen J. M. Bakker, Jos H. A. M. Kox, Harald S. Miedema, Sita Bierma-Zeinstra, Jos Runhaar, Cécile R. L. Boot, Allard J. van der Beek, Pepijn D. D. M. Roelofs

**Affiliations:** 1 Rotterdam University of Applied Sciences, Research Center Innovations in Care, Rotterdam, the Netherlands; 2 Department of Public and Occupational Health, Amsterdam University Medical Center – Amsterdam Public Health research institute, Amsterdam, the Netherlands; 3 Department of General Practice, Erasmus University Medical Center, Rotterdam, the Netherlands; 4 Department of Orthopaedics, Erasmus University Medical Center, Rotterdam, the Netherlands

**Keywords:** Nursing students, Dropout, Attrition, Sickness absence, Distress, Musculoskeletal complaints, Physical activity, Engagement, Work-related determinants, Cohort study

## Abstract

**Background:**

The shortage of nursing professionals is of growing concern. The causes of this include the demanding physical and mental workload, leading to a dropout of nurses that may start during their education. However, it is unclear to what extent nursing students already perceive a physical and mental workload leading to health problems during their nursing education and placement, and to what extent these health problems cause students to dropout from nursing education. Very few prospective cohort studies have investigated protective and risk factors in relation to dropout and retention among nursing students.

**Methods:**

Three cohorts of third-year nursing students will be followed for 2.5 years. Students will be enrolled from the Bachelor of Nursing program of the Rotterdam University of Applied Sciences. At baseline, students will receive a self-administered questionnaire. Primary outcome is dropout from nursing education and dropout from the nursing profession. Data on dropout from nursing education will be retrieved from the student administration on a yearly basis. Dropout from the nursing profession will be measured one year after graduation, using the self-reported questionnaire. Secondary outcomes are presenteeism and sick leave (during internship/work). In addition to student characteristics, the questionnaire asks about physical and mental internship/work characteristics, personal and behavioral factors, and experienced physical and mental burden.

Main aims of this study are to determine: 1) the prevalence and incidence rates of dropout, 2) the protective and risk factors, and early indicators of dropout, and 3) the interaction between these factors and the indicators.

**Discussion:**

Data analysis of a large, prospective cohort study with regard to determinants of dropout and retention of nursing students and newly graduated nurses is in progress. Findings emerging from this study can be used to develop a predictive model to identify the first indicators of dropout from nursing education and nursing profession, for which targeted interventions can be deployed.

## Background

In an aging population, a shortage of nurses poses a serious threat to the continuity and quality of health care. This shortage often results from increased demand combined with a declining number of new workforce entrants [1]. In the Netherlands, the number of registered nurses has decreased since 2013 [2] and there is a shortage of specialized nurses (i.e. emergency and intensive care, oncology, neonatology nurses) [3]. Moreover, the demand for nurse practitioners in the Netherlands is expected to double by 2028 [4].

The shortage of nursing professionals is also a growing concern in the European Union [5]. The European Commission Health workforce acknowledges that there is a significant employee turnover in some fields of health care due to the demanding working conditions [6]. Dropout of nursing students and the early exit of nurses starting their career contribute to this shortage. Research in Australia [7], the USA [8], Canada [9], the UK [10, 11], Finland [12], Ghana [13], Japan [14] and Sweden [15] has shown that, among nurses, physical and mental health problems can lead to dropout and early exit, and that this is a global problem.

The numbers of dropout differ between countries. For example, in the UK in 2015 the average dropout rate for student nurses at universities was ≥20% [16]. In Italy in 2011 the nursing students’ academic failure rates were 35–37% [17]. In the Netherlands, the dropout rate among nursing students increased slightly between 2005 and 2013 from 20.5% to 21.1%, respectively [18].

Dropout is a complex issue involving a wide range of factors. In Europe, two studies investigated the early exit of nurses and both reported that a considerable proportion of nurses considered giving up nursing [19, 20]. In 2003, the multinational NEXT study [19] showed that the proportion of participants considering leaving nursing (several times per month, or more often) ranged from 8.8–36.2% in the participating countries [19, 21]. In the RN4CAST study (a cross-sectional study including 12 European countries), the percentage of nurses that intended to leave their current job ranged from 19 to 49% [20]. In the LANE study, the career pathways in three cohorts of Swedish nursing students were prospectively followed for the first three years of their working life [22]. The intention to leave the profession one year after graduating ranged from 10 to 20% and was more common among younger nurses; in the 2002 cohort, about 2% of the participants had actually left the nursing profession within five years after graduation [22]. This indicates that the intention to leave does not necessarily lead to actual turnover.

The intention to leave nursing education or the nursing profession is associated with determinants of study burnout [23], job satisfaction, organizational commitment [24], job demands and work engagement [25]. In 2003 the determinants of stress, burnout and attrition in nursing students, and the relationships between these determinants, were measured in a prospective longitudinal cohort study; the results show that stress, burnout and attrition might be indirectly associated [26].

In the Netherlands, research among 11,000 healthcare employees (including 3057 nurses) revealed that many suffered from physical or mental health complaints due to work-related issues [27]. Nurses reported problems related to the locomotor system, severe fatigue, and feelings of frustration or burnout. In the latter study, half of the nurses reported to have visited a healthcare professional for physical problems, and one out of six for mental problems [27].

It is unclear to what extent (student) nurses already perceive these health problems during their nursing education and/or at the beginning of their career, and to what extent these health problems cause students to dropout from nursing education or their profession. Therefore, the SPRiNG (Studying Professional Resilience in Nursing students and Graduates) project was started. SPRiNG is a collaboration between Rotterdam University of Applied Sciences (RUAS), Erasmus University Medical Center, Amsterdam University Medical Center, and the Netherlands Institute for Health Services Research (NIVEL).

This article describes the protocol of the SPRiNG cohort study. The aim of this prospective study is to examine dropout and retention of nursing students during their education and/or at the start of their career, and the related protective and risk factors.

## Methods

### Study design

This is a prospective cohort study including three consecutive cohorts of third-year nursing students from RUAS, followed until one year after graduation.

### Setting

Rotterdam is the second largest city in the Netherlands, with ≥600,000 inhabitants. The RUAS has ≥36,000 students and offers a wide variety of programs in almost all educational sectors. The Bachelor of Nursing is their accredited four-year nursing educational program. According to the Netherlands Association of Universities of Applied Sciences (NAUAS) [18], the inflow of nursing student varies between the 15 Dutch universities of applied sciences and per year. In 2015 the Bachelor of Nursing program of RUAS had the largest inflow with 443 nursing students starting their first year. In 2016, RUAS had dropped to the eighth place, with 345 students.

In recent years, the majority of nursing students has failed to finish the program within four years. The graduation rate after five years of study among fulltime students dropped from 56.9% in 2007 to 39.8% in 2011. Students with a non-western migrant background had the lowest graduation rate, i.e. 33.3% in 2007 and 19.8% in 2011. Within the RUAS nursing program, dropout rates between 2002 and 2012 increased from 20 to 26.5%.

### Study population

**Fig. 1:**
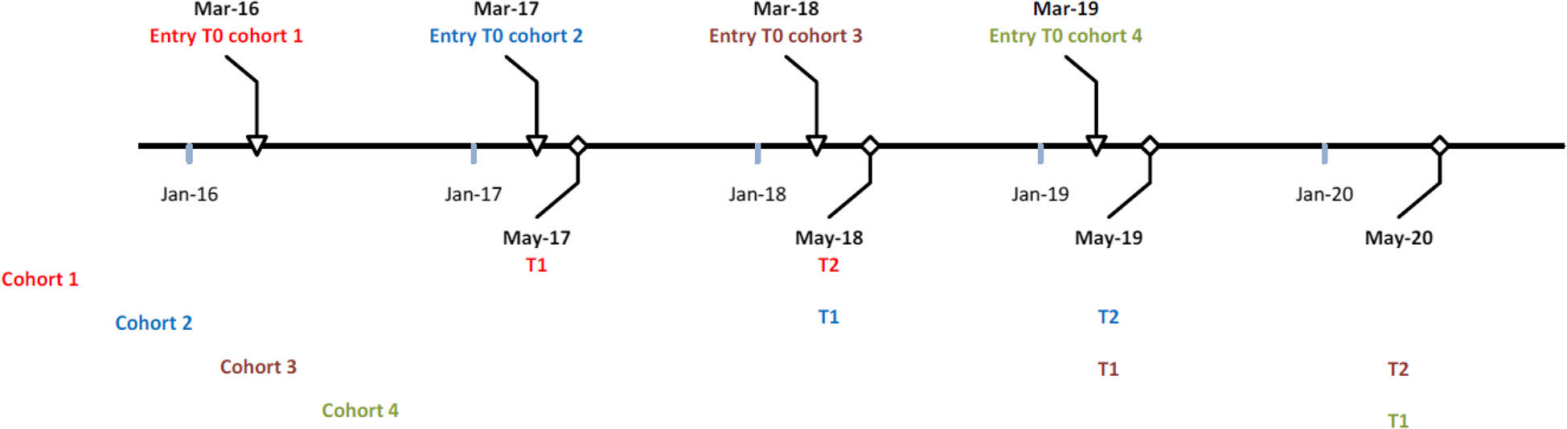
Timeline of the SPRiNG cohort study

For the present study, three cohorts of third-year nursing students will be followed for three years. They will receive a self-administered questionnaire in the third (t0) and fourth (final) year of their nursing study (t1), and again in their first year as a graduate nurse (t2). The first and the second cohort will be followed for three years and we plan to continue monitoring the third and fourth cohort (Fig. 1).

Participation by the nursing students is facilitated within the educational program, by offering questionnaires as part of the curriculum during lessons that address their professional development. They will be informed about the study before being approached for participation. Students can choose whether or not to make their data available for this research. All students who complete the questionnaire at t0 and give informed consent will be followed yearly.

Alumni and social networks will be used to restore lost contacts after the student has left the university. Non-respondents will be contacted by telephone to try and retrieve their job status.

**Fig. 2:**
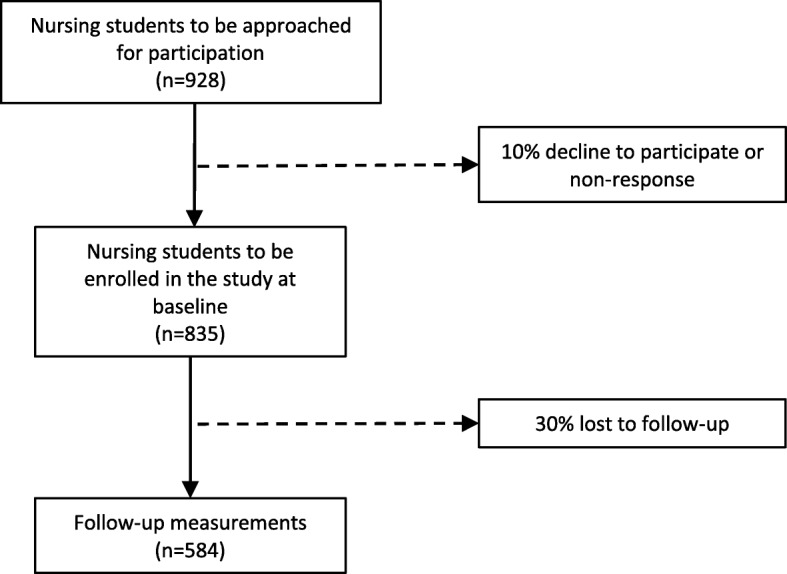
Flowchart of recruitment, study procedures and the expected response

The inclusion of students started in May 2016 (Fig. 2). Based on the response rate at t0 from the first two cohorts, and at t1 from the first cohort, we estimated the numbers expected to be included in this study.

### Primary outcome

The primary outcome is dropout from nursing education in the second half of the educational period and dropout from the nursing profession during the first year of their career. Dropout from education will be retrieved from the student administration on a yearly basis. In addition, one year after graduation (t2), dropout among working nurses will be measured using a self-reported questionnaire.

### Secondary outcomes

#### Sickness presenteeism

Presenteeism is defined as ‘Going to work despite judging one’s current state of health as such that sick leave should be taken’ and will be measured by the following question: ‘*Did it happen during your current internship/work that you have gone to internship/work despite the feeling that you really should have taken sick leave because of your state of health?*’ [28]. This is a translation of the question used in the original Dutch-language questionnaire ‘Healthy Working in Healthcare’ (i.e. *Gezond werken in de zorg*) [27].

#### Sick leave

Absenteeism due to illness will be measured by three questions included in the Netherlands Working Conditions Survey 2014 [29]: ‘*Have you ever been on sick leave during this academic year*? (‘Yes’/‘No’), ‘*How often have you been absent due to sickness*?’ (number of times), and ‘*How many days of work, all together, have you been on sick leave by estimation?*’ (number of days).

#### Absenteeism due to physical and mental health complaints

A question on absenteeism due to physical health complaints was taken from the Dutch Questionnaire on Experience and Evaluation of Work (VBBA) 1994 [30]. In 1999, the Commission Testing Affairs Netherlands (COTAN) of the Dutch Association of Psychologists (NIP) judged the VBBA to be good in terms of reliability and construct validity [30]. Five questions were used to measure physical health symptoms from neck, back and limbs; responses were scored on a 4-point Likert scale ranging from ‘always’ to ‘never’.

The Labour Monitor Municipalities (Arbomonitor Gemeenten) [31] will be used to measure absenteeism due to work pressure/work stress. The questions on absenteeism due to work pressure/work stress were developed in collaboration with AStri, an independent policy research agency in the field of work and income [31].

Six specific questions will be used to measure absenteeism due to mental health complaints, with response options: ‘Yes’/‘No’.

Professional support regarding physical health problems, mental health problems and social problems, will be measured with three items of the Healthy Working in Health Care questionnaire [27].

1) Physical: ‘*In the current internship/work period did you look for help regarding the previously mentioned physical complaints*?’ Response options are: ‘No’; ‘Yes, a general practitioner/company doctor’; ‘Yes, a physiotherapist’; or ‘Yes, another healthcare professional’.

2) Mental: ‘*In the current academic year did you visit a healthcare professional for mental help or support?’* Response options are: ‘No’; ‘Yes, a general practitioner’; ‘Yes, a psychologist/psychiatrist’; ‘Yes, a university counsellor’; or ‘Yes, another’.

3) Social: ‘*Do you have at this moment due to social problems like financial problems, housing problems etc. a referral to/contact with…?*’ Response options are: ‘No’; ‘Yes, a social worker; ‘Yes, a psychologist’; ‘Yes, a debt counsellor’; or ‘Yes, another’.

The composite questionnaire will include the secondary outcomes (sickness presenteeism and sickness absenteeism), and various general, physical and mental health items. Specific areas include: demographics, internship/work characteristics, personal and behavioural factors, and mental and physical health. Table 1 presents an overview of the instruments to be used for the measurements at t0, t1 and t2.

**Table 1:** Overview of the study outcomes and scales

Outcomes	Instrument and source
Primary outcome	
• dropout	(retrieved from student administration)
Secondary outcomes	
• presenteeism (during internship/work)	Sickness Presenteeism, Aronsson and Gustafsson, 2005 [28]
• sick leave (during academic year)	Sickness Absenteeism, NEA, Hooftman, Mars, 2015 [29]
Determinants/Potential predictors	
*Internship/work characteristics (general)*	
• decision latitude (skill discretion + decision authority) • psychological job demands • physical exertion • social support from supervisor • social support from co-workers	JCQ, Karasek, Brisson, 1998 [32]
*Internship/work characteristics (physical)*	
• lifting and bending	NEXT, Kümmerling, Hasselhorn, 2003 [33]
• monitor work	VBBA, van Veldhoven and Meijman, 1994 [30]
*Internship/work characteristics (mental)*	
• aggression and violence • bullying • slander	COPSOQ II, Pejtersen, Kristensen, 2010 [34]
• discrimination	NEA, Hooftman, Mars, 2015 [29]
*Personal and behavioral factors*	
• work engagement (vigor, dedication, absorption)	UWES-S, Schaufeli and Bakker, 2006 [37]
• occupational self-efficacy	Occupational Self-Efficacy Scale short version, Rigotti, Schyns, 2008 [39]
• work-family conflict, family-work conflict	WFC scale and FWC scale, Netemeyer, Boles, 1996 [35]
• physical activity	SQUASH, Wendel-Vos et al. 2003 [40]
*Experienced physical burden*	
• musculoskeletal symptoms	DMQ, Hildebrandt, 2001 [46]
• use of support for physical health problems	‘Gezond werken in de zorg’ [Healthy Working in Healthcare] questionnaire, Bronkhorst, ten Arve, 2014 [27]
*Experienced mental burden*	
• distress	Distress Screener, Braam, van Oostrom, 2009 [41]
• need for recovery	NFR scale, Van Veldhoven and Broersen, 2003 [44]
• use of support for mental health problems	VBBA, van Veldhoven and Meijman, 1994 [30]

The digital questionnaire ensures standardized responses to questions and eliminates out-of-range responses. As backup, a paper version of the questionnaire will be available for students. Whenever available, validated constructs will be used. If necessary, questions are rephrased to fit the target group. For example, when the original question is about paid work, it is rephrased as ‘internship/work’, to address a student.

### Population characteristics

Questions on respondent characteristics include: gender (male/female), age (years), body height and weight (BMI), educational background (secondary vocational education/higher professional education/university), nursing educational pathway (fulltime, part-time, in-service), ethnicity (Dutch/western migrant/non-western migrant), Dutch as first language (yes/no), and housing circumstances (living with parents or caregivers/ living on one’s own/ living on one’s own with kids/living on one’s own with partner/living on one’s own with partner and child(ren)). Information on these characteristics will be collected at t0 only.

#### Internship/work-related physical and psychosocial risk factors

For these determinants, six subscales of the validated Dutch version of the Job Content Questionnaire (JCQ) will be used [32], i.e. skill discretion, decision authority, psychological job demands, physical exertion, social support from supervisor, and social support from co-workers. The JCQ measures the physical and psychological characteristics of an imbalance between job demands and resources within an organization. Four self-formulated questions regarding feedback and guidance from the instructor and colleagues will be added (‘*My instructor gives me constructive feedback’*; ‘*My colleagues give me constructive feedback’; ‘When I got stuck in my learning process I have somebody to discuss this with’*, and ‘*My instructor has enough time for my guidance’*). Responses are on a 4-point Likert scale ranging from ‘totally disagree’ to ‘totally agree’.

#### Lifting and bending, visual display units work

For these determinants, 13 questions related to lifting and bending were taken from the NEXT study [33]. A scale assessing lifting and bending was developed by the NEXT Study Group on the basis of own validity measurements including pre-tests, in order to quantify the specific physical demands in the nursing profession. The scales were translated from English into Dutch and back to English by four independent native English/Dutch speakers, in order to validate the scales in Dutch language for the SPRiNG study.

Two questions on Visual Display Unit work were taken from the Dutch Questionnaire on the Experience and Evaluation of Work (VBBA) [30].

#### Aggression and violence, bullying, slander, discrimination

Three single-item questions on aggression and violence, bullying, and slander were taken from the second version of the Copenhagen Psychosocial Questionnaire (COPSOQ II) [34]. The Dutch translation was obtained from the Healthy Working in Healthcare questionnaire [27] and was used among healthcare professionals, including nurses. Discrimination will be measured by one question from the Netherlands Working Conditions Survey 2014 [29].

#### Work-family conflict, family-work conflict

Work-family conflict and family-work conflict will be measured using the Netemeyer, Boles scales [35]. These authors defined work-family conflict as: “*A form of interrole conflict in which the general demands of time devoted to and strain created by the job interfere with performing family-related responsibilities*”; and family-work conflict as *“A form of interrole conflict in which the general demands of time devoted to and strain created by the family interfere with performing work-related responsibilities.”* [35].

### Personal and behavioral factors

#### Work engagement

Schaufeli & Bakker [36] defined work engagement as *“…a positive, fulfilling, work-related state of mind that is characterized by vigor, dedication, and absorption*.” Work engagement will be measured with the 9-item short version of the Utrecht Work Engagement Scale-Short (UWES-S) [37].

#### Occupational self-efficacy

Occupational self-efficacy refers to the confidence a worker has in his/her perceived ability to successfully perform job tasks [38]. This will be measured with the six-item short version of the Occupational Self-efficacy scale [39].

#### Physical activity

The Short QUestionnaire to ASses Health enhancing physical activity (SQUASH), will be used to measure physical activity [40]. SQUASH is a fairly reliable (*r* = 0.58) and reasonably valid (*r* = 0.45) questionnaire to measure physical activity. SQUASH will assess the activities during a regular week in the past month with regard to the duration, frequency, and intensity of leisure time activities, household activities, activity at work and school, and commuting activities [40].

### Mental and physical health

#### Distress

To measure non-specific distress we will use the Distress Screener, which comprises three items of the 4DSQ distress subscale. The 4DSQ is a self-report 50-item questionnaire that measures non-specific distress, depression, anxiety and somatization. For the purpose of this study a short questionnaire, and a sensitive instrument able to detect early signs of mental health problems, are needed. The Distress Screener (developed for early identification of non-specific distress) has three items; we will use a cut-off point > 4 to detect moderate distress [41]. The Distress Screener is a valid instrument for early identification of distress in employees on sick leave as well as for non-sick listed employees at risk of future mental sickness absence [42].

#### Need for recovery

Need for recovery has been conceptualized as the experience of accumulating work-induced fatigue and is an early indicator of risk of depression [43]. The Need for Recovery after work (NFR) scale [44] is a part of the VBBA [30]. The NFR scale consists of 11 dichotomous items (‘Yes’/‘No’) and has good reliability, concurrent validity and sensitivity to change [45].

#### Musculoskeletal symptoms

Questions related to health (particularly musculoskeletal symptoms) from the Dutch Musculoskeletal Questionnaire (DMQ) will be used [46]. The phrasing of these questions regarding prevalence is comparable with the Nordic Questionnaire on Musculoskeletal Disorders [47], including definition of the areas of the body using a mannequin. The DMQ enables global assessment of physical workload and other potentially hazardous working conditions. Most indices show significant associations with low back and/or neck-shoulder symptoms; therefore, these indices can be used as one of the means to identify risk groups [46]. In the t0 questionnaire this will be asked two times (for the current training and the previous practical training), since most third-year students do a practical training in the first semester of the academic year and another in the second semester. This type of retrospective measurement will give an indication about the accumulation of musculoskeletal symptoms.

#### Expectations regarding the nursing program and nursing profession

Expectations about the nursing program and profession will be measured using seven self-formulated questions: ‘*My internship/work corresponds with my expectations of the nursing profession’; ‘My internship/work corresponds with what I learn at the university’; ‘I expect to stay working in the healthcare sector after graduation’; ‘I expect to finish nursing school with a diploma’; ‘I expect to stay working as a nurse after graduation’; ‘I am expecting a study delay’*; and ‘*I consider to quit my study’*. Answers are rated on a 10-point Likert scale ranging from ‘definitely not’ to ‘definitely yes’.

### Data handling and statistical analyses

Key aims of this study are to determine: 1) incidence rates of dropout, 2) protective and risk factors, and early indicators of dropout, and 3) interactions between these factors and indicators.

#### Data handling

Data will be collected using Limesurvey (Version 2.06lts Build 160,524). Data will be exported to a secured SPSS database for management and analyses. To avoid potential conflict of interest, the principle researchers will be blinded from any results that can relate data back to the individual respondents; therefore, this work will be done by independent researchers. Personal data will be extracted from the dataset before analysis takes place. Analyses will be carried out using IBM SPSS Statistics, version 24 or higher (IBM Corp., NY, USA).

#### Preliminary analysis and transformation of variables

First, for each cohort the differences in demographics (age, gender, educational level at entrance, and study route) and the primary outcome ‘dropout’ between students included and not included (non-responders) in the cohort will be compared. For students in the cohort, data from the student administration will be used.

Second, descriptive statistics of outcomes and determinants will be provided and quantitative variables will be depicted graphically using histograms and normal probability plots.

#### Assessment of prevalence and incidence

In the study population, point prevalence will be estimated for mental and physical health problems, absenteeism, presenteeism, and sick leave at baseline (t0), after one year (t1), and one year after graduation (t2), in order to characterize the cohort. Incidence in the study population will be calculated for mental and physical health problems, absenteeism, presenteeism, and dropout after one year (t1) and also one year after graduation (t2).

#### Regression and covariate adjustment

To relate dropout and retention to potential determinants and covariates, regression analyses will be conducted. First we will analyse the univariate relationships between all potential determinants (protective and risk factors, and early indicators) and outcomes (dropout, absenteeism, presenteeism, retention). Then, a multivariate model will be constructed for all determinants with an association of *p* < 0.05. To study the relation between one or more independent variables with the continuous dependent variables (absenteeism, presenteeism), linear regression analyses will be used. Logistic regression analyses will be conducted to study the relation with dependent dichotomous variables (intention to leave nursing school or profession, actual dropout).

A latent class analysis [48] will be performed to identify subgroups. This analysis will focus on the relations between individual participants, instead of the relations between variables. Response patterns can be revealed that might be distinctive for a subgroup and will differ from response patterns in other subgroups.

#### Missing data

We expect to have follow-up data (determinants and secondary outcomes) from at least 80% of all students. Primary outcome data (dropout) will be available for all students from the student administration. We will anticipate to the possible missing values (MCAR & MAR). For statistical analysis techniques will be used that are robust for missing values (modelling to collected data) and sensitivity analyses will be performed on multiple imputed data sets [49].

## Discussion

This study will provide information on 1) the prevalence and the incidence rates of dropout, 2) the protective and risk factors, and early indicators of dropout, and 3) the interactions between these factors and indicators. This article describes the protocol and methodology of the study.

### Strengths and limitations

Few longitudinal studies are available on nursing students and recently graduated nurses. The actual shortage of nurses necessitates the prevention of avoidable dropout. Implementation of effective preventive interventions with regard to physical and mental resilience may help to ensure a sufficient number of nurses, which is an essential condition to guarantee adequate quality of care. Therefore, we need to know which determinants play an important role and which of these determinants are modifiable.

A potential limitation of the present study is that respondents originate from RUAS only. To generalize our results to the national population of nursing students and new graduates, we will investigate to what extent the population characteristics of our Rotterdam sample differ from the national population of nursing students and new graduates, as available through NAUAS [18].

The findings of this study can be used to develop a predictive model that identifies early signals for dropout from nursing education and nursing profession, for which potentially targeted interventions can be deployed. Plans within the SPRiNG project include exploring yet unknown reasons for dropout through qualitative research, systematic reviews of effective preventive interventions, and testing of the most feasible interventions in a pilot study. These steps will provide an additional toolbox with targeted interventions that can be implemented in nursing education or nursing practice to prevent dropout.

## Abbreviations

4DSQ: Four-Dimensional Symptom Questionnaire
BMI: body mass index
CBS: Centraal Bureau voor de Statistiek [Statistics Netherlands]
COPSOQ: Copenhagen Psychosocial Questionnaire
COTAN: Commissie Testaangelegenheden Nederland [Commission Testing Affairs Netherlands]
DMQ: Dutch Musculoskeletal Questionnaire
FWC: Family-work conflict
JCQ: Job Content Questionnaire
NAUAS: Netherlands Association of Universities of Applied Sciences
NEA: Nationale Enquête Arbeidsomstandigheden [National Questionnaire Working Conditions]
NEXT: Nurses’ Early Exit Study
NFR: Need For Recovery
NIP: Nederlands Instituut van Psychologen [Dutch Association of Psychologists]
NIVEL: Nederlands instituut voor onderzoek van de gezondheidszorg [Netherlands institute for health services research]
NWO: Netherlands Organisation for Scientific Research
RUAS: Rotterdam University of Applied Sciences
SPRiNG: Studying Professional Resilience in Nursing students and Graduates
SQUASH: Short QUestionnaire to ASses Health enhancing physical activity
UWES-S: Utrecht Work Engagement Scale – Short
VBBA: Vragenlijst Beleving en Beoordeling van de Arbeid [Questionnaire Experience and Evaluation of Work]
VDU: Visual display Unit
WFC: Work-family conflict
